# Acute Infectious Purpura Fulminans Complicated by Bacterial Translocation after Rectal Cancer Surgery: A Case Report

**DOI:** 10.3390/medicina60040644

**Published:** 2024-04-17

**Authors:** Ryo Nakanishi, Heita Ozawa, Naoyuki Toyota, Minori Mise, Ritsuto Akutsu, Shin Fujita

**Affiliations:** Department of Colorectal Surgery, Tochigi Cancer Center, 4-9-13 Yohnan, Utsunomiya 320-0834, Japan; heiozawa@tochigi-cc.jp (H.O.); naoyuki.toyota@gmail.com (N.T.); tsc.ninja400@gmail.com (M.M.); riakutsu@tochigi-cc.jp (R.A.); sifujita@tochigi-cc.jp (S.F.)

**Keywords:** rectal cancer, acute infectious purpura fulminans, bacterial translocation

## Abstract

The patient was a man in his 80s who had undergone laparoscopic anterior resection for rectal cancer. Bowel obstruction occurred on the third postoperative day but improved with a decompression tube by the fifth postoperative day. A high fever (in the 38 °C range) was also observed. Blood culture tests detected two sets of the gram-negative bacilli *Klebsiella aerogenes* within 24 h of collection. On the seventh postoperative day, the patient subsequently went into septic shock with disseminated intravascular coagulation (DIC). On the eighth postoperative day, the fingertips and toes became black, and the palms and dorsal surfaces of both feet were dark purple due to peripheral circulatory failure. This suggested acute infectious purpura associated with sepsis (acute infectious purpura fulminans (AIPF)). Intensive care was provided; however, the necrosis of both middle fingers worsened, both middle fingers were gangrenous, and the patient died on the thirtieth postoperative day. AIPF is rarely reported, especially in early-onset cases after elective surgery. We encountered a rare complication of bacterial translocation from postoperative bowel obstruction, leading to AIPF.

## 1. Background

Purpura fulminans (PF) is characterized by fever, shock, multiple organ failure, and rapidly progressive and spreading peripheral purpura and skin necrosis. The infection-induced form is called acute infectious purpura fulminans (AIPF) [1]. The reported mortality rates are frequently greater than 30%, and limb amputation is ultimately required even for cases that can be salvaged [2]. AIPF after elective abdominal surgery is rare. This is a report of a case of AIPF due to sepsis after elective surgery for rectal cancer and a review of the literature.

## 2. Case Presentation

**Figure 1:**
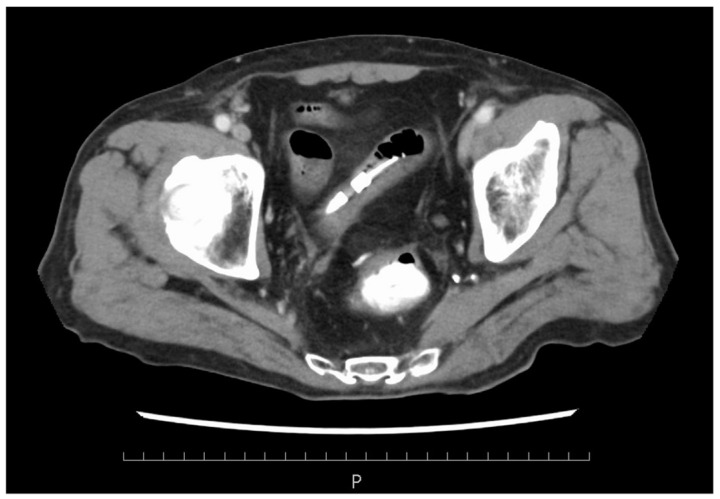
Abdominal contrast-enhanced CT showed no obvious anastomotic leakage, and the bowel obstruction had improved.

**Figure 2:**
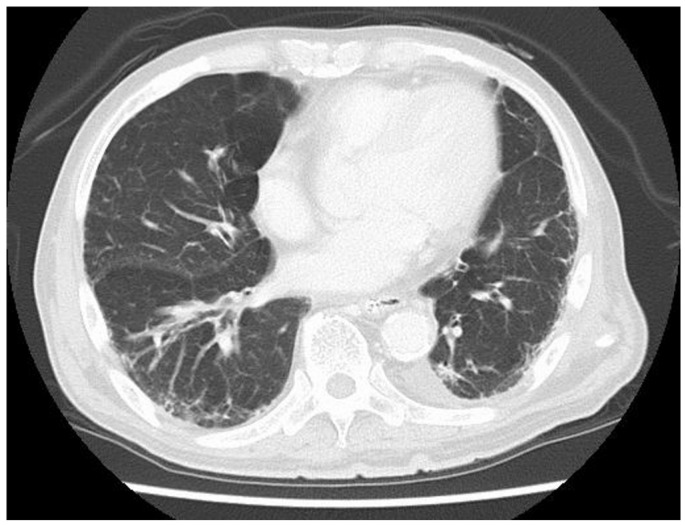
Thoracic CT showed mild aspiration pneumonia without exacerbation of interstitial pneumonia.

**Figure 3:**
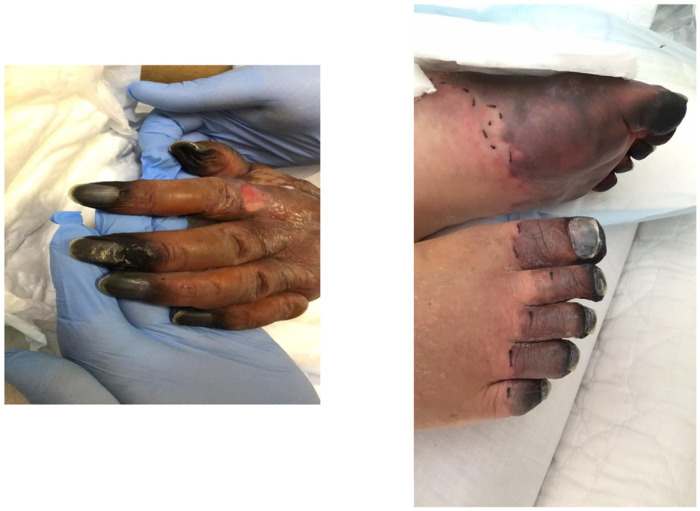
The tips of both hands and feet turned black, and the palms and dorsal surfaces of both feet were dark purple due to peripheral circulatory failure.

The patient was a man in his 80s. He had pre-existing hypertension, interstitial pneumonia, and lung cancer (pT1cN0M0 and pStageIA3). He had a height of 158 cm, a weight of 53.5 kg, a BMI of 21.43 kg/m^2^, and ASA-PS class III. He could also independently perform activities of daily living. Blood tests showed mild renal dysfunction with a Cr of 1.1 mg/dL and an eGFR of 44.4, but the echocardiogram showed only mild cardiac hypertrophy and no wall motion problems. His operative tolerance was judged to be fine. For preoperative mechanical bowel preparation, 24 mg of sennoside was administered two days before surgery, and 34 g of magnesium citrate was administered the day before surgery. Kanamycin sulfate (2 g/day) and metronidazole (1 g/day) were administered orally in four divided doses a day before surgery. One gram of cefmetazole was administered for prophylaxis within 30 min before surgery, and additional doses were administered every 3 h during surgery. Postoperative antibiotics were administered every 12 h and discontinued on the first postoperative day. The patient underwent laparoscopic anterior resection of rectal cancer (cT2N0M0, cStage I). The duration of surgery was 2 h 40 min, and the estimated blood loss was 5 mL. The patient was anesthetized for 3 h 48 min, and pneumoperitoneum was maintained for 2 h 21 min. A transfusion volume of 1500 mL was prepared, but no blood transfusion was administered. On the first postoperative day, no obvious abdominal distension or fever was observed, and clear liquid intake was initiated. Blood tests showed mild renal dysfunction with a Cr of 1.22 mg/dl and a CRP of 3.9 mg/dL. The patient passed without obvious abdominal distension or fever on the second postoperative day. On the third postoperative day, vomiting was observed, and an abdominal radiograph showed an air–fluid level, which led to the diagnosis of ileus and the insertion of an ileus tube. The nausea and vomiting improved, and abdominal decompression was observed. On the fifth postoperative day, gastrointestinal angiography showed that the gastrografin had flowed to the colon, and the drainage volume of the ileus tube was low at 530 mL. The ileus was considered to be mild, and oral intake was scheduled to resume on the sixth postoperative day. However, he developed a cough in the morning and a fever (in the 38 °C range) in the evening of the sixth day, and his SpO_2_ dropped to 92%. Therefore, a thoracoabdominal contrast-enhanced CT (computer tomography scan (CT)) was performed. Cough and dyspnea were observed in the early morning of the fifth postoperative day, and meropenem 0.5 g was initiated because of the high fever (38.5°). Thoracoabdominal contrast-enhanced CT showed that the bowel obstruction had improved and there was no obvious anastomotic leakage (Figure 1). Mild pneumonia was observed, but there was no exacerbation of interstitial pneumonia (Figure 2). Therefore, MEPM was started as an empiric therapy because of the fever and advanced age. Blood culture tests detected two sets of the gram-negative bacilli *K. aerogenes* within 24 h of collection. MEPM was sensitive to *K. aerogenes*. The bacteria detected were not endemic to the oral cavity, and there was no history of vomiting. Aspiration pneumonia could not be confirmed on the seventh postoperative day. His respiratory status worsened, and his SpO_2_ was approximately 94% even with 10 L of oxygen. His respiratory rate exceeded 15 breaths per minute. His arterial partial pressure of oxygen was 74 mmHg, and he became disoriented. His systolic blood pressure was approximately 80 mmHg. Septic shock was diagnosed, and the patient was placed on a ventilator in the intensive care unit. Blood tests revealed decreased platelet counts and prolonged coagulation, which were associated with DIC (PT 15.8 s, APTT 53.1 s, platelet counts 44,000/µL, d-dimer 238 µg/mL). Despite high doses of noradrenaline and vasopressin, his blood pressure remained approximately 70 mmHg, and he was continuously oliguric and unresponsive to fluid infusion. On the morning of the eighth postoperative day, purpura appeared on both fingers and toes. The purpura further extended to the palms, dorsal surfaces, wrist joints of both upper limbs, and the plantar surfaces of the lower limbs. Finally, the tips of both fingers became black (Figure 3). Therefore, the patient was diagnosed with AIPF associated with sepsis. Polymyxin B-immobilized fiber column direct hemoperfusion (PMX-DHP) and continuous hemodiafiltration dialysis are difficult to initiate at an early stage because of prolonged hypotension. As blood pressure gradually increased with continued DIC treatment and fluid replacement, continuous hemodiafiltration was initiated on the eleventh postoperative day. The patient was weaned from continuous hemodiafiltration on the sixteenth postoperative day and from ventilator management on the seventeenth postoperative day due to improvement of his general condition. The black necrosis of both fingers was mummified, and the symptoms resolved. However, the patient and his family did not wish to undergo amputation because of progressive necrosis of the left leg. On the twenty-sixth postoperative day, the patient and his family agreed that they did not want active treatment. The patient died on the thirtieth postoperative day due to a soft tissue infection and a worsening general condition.

## 3. Discussion

PF is classified according to its cause into congenital and acquired coagulation abnormalities (e.g., PC deficiency), acute infectious type, and idiopathic type. The acute infectious type is called AIPF and is considered a rare complication of septic shock [1,2]. It was first described by Guelliot et al. in 1844 [1]. *Neisseria meningitidis* is the most common causative organism of AIPF, followed by *Streptococcus pneumoniae* and Haemophilus influenzae [3]. Bacteria of the genera Streptococcus [4,5], *Staphylococcus aureus* [6], and Capnocytophaga canimorsus have also been reported [7]. Conversely, *Klebsiella aerogenes* in the Enterobacteriaceae are rarely associated with AIPF. 

The pathogenesis of AIPF remains unclear, but necrotizing vasculitis is speculated to be induced by reduced blood flow, DIC, and cytokine induction by endotoxemia, leading to thrombotic hemorrhagic necrosis of the skin [2]. In the present case, postoperative paralytic ileus caused disturbance of the intestinal microbiota and breakdown of the intestinal mucosal barrier; the intestinal bacterium *Klebsiella aerogenes* entered the bloodstream, leading to septic shock from bacterial translocation (BT). The diagnosis of BT is difficult and requires (1) ruling out other proven sources of infection; (2) positive enterolymph node cultures or positive blood or ascites fluid cultures with identified bacterial species identical to those in the intestinal tract; and (3) confirmation of bacteria in the intestinal mucosa under a microscope [8]. However, the last two are rarely carried out in practice, and direct clinical diagnosis is limited to special circumstances [8]. Positive blood cultures and measurements of endotoxin concentrations were also used as indirect findings. In this case, sepsis developed during ileus, and multiple blood culture tests detected the enterobacterium *Klebsiella aerogenes*, which led to the diagnosis of BT. A PubMed search was performed using the keywords ”bacterial translocation” and “acute infectious purpura fulminans” for the period 1950–2023, but no cases were reported. A search of Ichushi-Web for ”bacterial translocation” and “acute infectious purpura fulminans” revealed three reported cases [9,10,11]. Only one case [10] was associated with postoperative BT (Table 1). The patient had a history of multiple bowel obstructions with a background of Crohn’s disease, and BT occurred after bowel obstruction repair, which is quite different from the course of our case. In addition, two cases of AIPF after elective surgery have been reported. The first case was AIPF after surgery for sigmoid colon cancer with abscess formation in a patient with diabetes mellitus [12], and the other case was AIPF after cholecystectomy for cholecystitis with preoperative cholangitis [13]. The courses of both cases were completely different from ours. A PubMed search for the period between 1985 and 2023 using the keywords “rectal cancer” and “purpura fulminans” did not reveal any corresponding cases. Therefore, we consider this to be the first report of AIPF following BT after elective surgery for rectal cancer. The lesson learned from this case is that postoperative bowel obstruction after gastrointestinal surgery is relatively common, and it can lead to AIPF.

Early diagnosis and treatment of AIPF are important because the prognosis of AIPF is generally poor [2], and treatment of the causative infection and DIC is the primary focus. One intervention for our patient was the early introduction of PMX-DHP. PMX-DHP also improves hypotension and tachycardia by adsorbing endogenous cannabinoids, which are thought to be involved in the pathogenesis of septic shock [14]. Recent evidence of endotoxin adsorption therapy for sepsis is negative, but a multicenter, retrospective study conducted in Japan involving patients with septic shock showed that PMX-DHP was effective when performed earlier [15]. In this case, PMX-DHP should have been introduced earlier.

## 4. Conclusions

Herein, we report a case of AIPF triggered by BT due to bowel obstruction after elective laparoscopic high anterior resection of rectal cancer. This is the first case of AIPF due to bacterial translocation following elective rectal cancer resection surgery, and it is a complication that any surgeon may experience.

## Figures and Tables

**Table 1:** Previous reports of AIPF resulting from BT.

No	Author	Year	Patient	Bacteria Detected	Turning
1	Shibuya [9]	2009	50-year-old male	*Citrobacter freundii*	Death
2	Kasai [10]	2018	40-year-old female	*Citrobacter freundii*	Survival
3	Yamamura [11]	2022	66-year-old male	*Klebsiella pneumonia*	Death
			73-year-old male	*Citrobacter freundii*	Death
4	Our case		80-year-old male	*Klebsiella aerogenes*	Death

## Data Availability

All data generated or analyzed in this study are included in the published article.
